# Intoxication due to *Papaver rhoeas* (Corn Poppy): Five Case Reports

**DOI:** 10.1155/2015/321360

**Published:** 2015-05-12

**Authors:** Yahya Kemal Günaydın, Zerrin Defne Dündar, Bora Çekmen, Nazire Belgin Akıllı, Ramazan Köylü, Başar Cander

**Affiliations:** ^1^Department of Emergency Medicine, Konya Training and Research Hospital, Konya, Turkey; ^2^Department of Emergency Medicine, Faculty of Medicine, Necmettin Erbakan University, Konya, Turkey

## Abstract

*Introduction*. In this paper, we aimed to present five *Papaver rhoeas* intoxication cases, which is very rare in the literature. *Case 1*. A 35-year-old female patient was admitted to our emergency room with the complaints of nausea, restlessness, and dyspnea developing 3 hours after eating *Papaver rhoeas*. On physical examination, her general condition was moderate; she was conscious and the vital findings were normal. The pupils were myotic. She was transferred to the toxicology intensive care unit as she experienced a generalized tonic clonic seizure lasting for three minutes. *Case 2*. A 41-year-old female patient was brought to our emergency room by 112 ambulance as she had contractions in her arms and legs, unconsciousness, and foam coming from her mouth two hours after *Papaver rhoeas* ingestion. On physical examination, she was confused, the pupils were myotic, and she was tachycardic. Arterial blood gases analysis revealed lactic acidosis. *Case 3*. A 38-year-old female patient was admitted to our emergency room with complaints of nausea and vomiting two hours after ingestion of *Papaver rhoeas*. Her physical examination and tests were normal. *Case 4*. A 34-year-old male patient was admitted to our emergency room with complaints of numbness and loss of power in his arms and legs one hour after *Papaver rhoeas* ingestion. He was hospitalized at the toxicology intensive care unit for follow-up and treatment. Dyspnea and bradycardia developed on the follow-up. The oxygen saturation without oxygen support was 90%. ECG revealed sinus bradycardia. The cardiac enzymes did not increase. *Case 5*. A 42-year-old female patient was brought to our emergency room by 112 ambulance with contractions in her arms and legs and unconsciousness two hours after *Papaver rhoeas* ingestion. On her physical examination, she was confused and the pupils were myotic. Arterial blood gases analysis revealed lactic acidosis. *Conclusion*. All patients were followed up for a few days and then discharged from the hospital with recovery. Unconscious consumption of *Papaver rhoeas* leads to a clinical condition resembling morphine intoxication, CNS depression, and epileptic seizures.

## 1. Introduction

**Figure 1:**
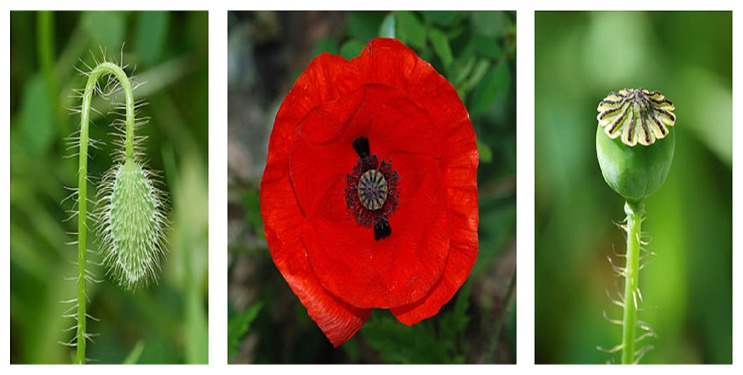
https://www.wikipedia.org/: a poster showing three different stages of a common poppy flower (*Papaver rhoeas*): bud, flower, and fruit (capsule) [1].

*Papaver rhoeas* is a plant which spontaneously grows in fields and grasslands and its flowers are in the shape of four blood red pots [1, Image 1]. It is in the benzylisoquinoline group of opium alkaloids and contains rhoeadine alkaloid. It does not lead to addiction [2, 3]. The plant is used for treating cough or sleep disorder or as food among the people (see Figure 1). It may lead to clinical conditions such as nausea, vomiting, confusion, seizures, myosis, arrhythmia, and morphine intoxication-like findings [2, 4–6].

In this paper, we aimed to present five* Papaver rhoeas* intoxication cases, which is very rare in the literature.

## 2. Cases

### 2.1. Case 1

A 35-year-old female patient was admitted to our emergency room with the complaints of nausea, restlessness, and dyspnea developing 3 hours after eating* Papaver rhoeas*. Approximately half a kilogram has been eaten from baked* Papaver rhoeas*. On physical examination, her general condition was moderate; she was conscious and the vital findings were normal. The pupils were myotic. Other system examinations, liver function test, renal function test, cardiac troponins, electrolytes, complete blood cell counts, electrocardiography (ECG), and computed tomography of the brain were normal. Urine toxicology screen was normal. This table describes any substance to be detected. She was transferred to the toxicology intensive care unit as she experienced a generalized tonic clonic seizure lasting for three minutes. Only supportive treatment was provided for this patient. The patient was followed up for two days and then discharged from the hospital with recovery.

### 2.2. Case 2

A 41-year-old female patient was brought to our emergency room by 112 ambulance as she had contractions in her arms and legs, unconsciousness, and foam coming from her mouth two hours after* Papaver rhoeas* ingestion. Approximately 250 grams has been eaten from baked* Papaver rhoeas*. On physical examination, she was confused, the pupils were myotic, and she was tachycardic. The arterial blood pressure was 90/60 mmHg, the plasma glucose was 112 mg/dL, and the oxygen saturation was 95%. ECG revealed sinus tachycardia. Arterial blood gases analysis revealed lactic acidosis. Liver function test, renal function test, cardiac troponins, electrolytes, complete blood cell counts, and computed tomography of the brain were normal. Urine toxicology screen was normal. This table describes any substance to be detected. Only supportive treatment was provided for this patient. The patient was followed up for two days and then discharged with recovery.

### 2.3. Case 3

A 38-year-old female patient was admitted to our emergency room with complaints of nausea and vomiting two hours after ingestion of* Papaver rhoeas*. Approximately 250 grams has been eaten from baked* Papaver rhoeas*. Her physical examination was normal. Liver function test, renal function test, cardiac troponins, electrolytes, complete blood cell counts, and ECG were normal. Urine toxicology screen was normal. This table describes any substance to be detected. The patient was followed up for one day and then discharged with recovery.

### 2.4. Case 4

A 34-year-old male patient was admitted to our emergency room with complaints of numbness and loss of power in his arms and legs one hour after* Papaver rhoeas* ingestion. Approximately half a kilogram has been eaten from baked* Papaver rhoeas*. His physical examination and neurological examination were normal. Liver function test, renal function test, cardiac troponins, electrolytes, complete blood cell counts, electrocardiography (ECG), and computed tomography of the brain were normal. Urine toxicology screen was normal. This table describes any substance to be detected. He was hospitalized at the toxicology intensive care unit for follow-up and treatment. Dyspnea and bradycardia developed on the follow-up. The oxygen saturation without oxygen support was 90%. ECG revealed sinus bradycardia. The cardiac enzymes did not increase. The cholinesterase level was tested in order to eliminate organophosphate intoxication considering that the* Papaver rhoeas* he had eaten contained organophosphate as he developed dyspnea and bradycardia. The cholinesterase level was normal. Only supportive treatment was provided for this patient. All complaints improved and laboratory tests were normal, and he was then discharged from the hospital.

### 2.5. Case 5

A 42-year-old female patient was brought to our emergency room by 112 ambulance with contractions in her arms and legs and unconsciousness two hours after* Papaver rhoeas* ingestion. Approximately 250 grams has been eaten from baked* Papaver rhoeas*. Approximately 250 grams from cooking* Papaver rhoeas* was eaten. On her physical examination, she was confused and the pupils were myotic. Arterial blood pressure was 110/70 mmHg, plasma glucose was 128 mg/dL, and the oxygen saturation was 98%. Arterial blood gases analysis revealed lactic acidosis. Computed tomography of the brain and liver function test, renal function test, cardiac troponins, electrolytes, and complete blood cell counts were normal. Urine toxicology screen was normal. This table describes any substance to be detected. Only supportive treatment was provided for this patient. The patient was followed up for one day and then discharged with recovery.

## 3. Discussion

There is little data available in the literature about* Papaver rhoeas* intoxication. We could not find studies on the subject, except for a few case reports, animal studies, and agricultural studies. Our series of five cases is the most comprehensive study in the literature. Therefore, it is quite significant.

Chemical studies have shown that the* Papaver rhoeas* extract is composed of rhoeadine, rhoeadic acid, papaveric acid, rhoeagenine, and anthocyanins [2, 5, 7, 8].* Papaver rhoeas* is a commonly found plant in our country. It is used as a medicinal plant [3].* Papaver rhoeas* is found in Iran and other countries of the world. It is used for treating diarrhea, cough, and sleep disorders and for analgesia-sedation purposes. It is also used for reducing opioid abstinence symptoms [2]. It has been reported to be used for treatment of intestinal and urinary irritation, bronchitis, pneumonia, and febrile diseases with rash [4]. However, none of our patients used* Papaver rhoeas* for treatment. They all used it for its good taste.

The* Papaver rhoeas*-induced allergic dermatitis case reported by Gamboa et al. [9] is among the rare cases in the literature. In another study, it was concluded that the* Papaver rhoeas* extract could heal abstinence syndrome, in addition to morphine abstinence syndrome in rats with morphine addiction [10]. In another study in rats,* Papaver rhoeas* was shown to reduce postural behaviors, locomotor activities, and analysis movements, in addition to having a sedating effect [11]. In the case report of Koçak et al. [6], central nervous system depression findings developing after eating* Papaver rhoeas* were reported.

The abovementioned rare clinical studies and case reports support the findings such as seizures, myosis, confusion, and CNS depression that were found in our case. We believe that this clinical situation is caused by* Papaver rhoeas* morphine-like effects, because previously phytochemical investigation of* Papaver rhoeas* has shown the presence of alkaloids, such as rhoeadine, allotropine, and coptisine. These alkaloids have dopaminergic antagonists and neuroleptic effects [5, 12, 13].

## 4. Conclusion

Unconscious consumption of* Papaver rhoeas* leads to a clinical condition resembling morphine intoxication, CNS depression, and epileptic seizures.* Papaver rhoeas* intoxication should be kept in mind in patients who present to the emergency room with altered state of consciousness or widespread neurological symptoms. Education and information activities should be emphasized so that* Papaver rhoeas* and similar plants are not used unconsciously.
